# To the Editors of the Medical and Physical Journal

**Published:** 1803-02-01

**Authors:** Ralph Blegborough

**Affiliations:** No. 330, Oxford Street


### *To the Editors of the Medical and Phyſical Journal.*

GENTLEMEN,

SOME time ago I had the pleasure of laying before the readers of your valuable Journal, the Case of John Rouneberg, a paralytic patient, who was sent to me by the late Dr. Garnett, from the Mary-le-bone Dispensary. I then stated, that he had received much benefit from six applications of the AIR-PUMP VAPOUR-BATH; and that I could not, at that early period, have omitted noticing the case, without calling in question the opinion of the Doctor.

The patient, however, gave me the slip, and got a situation somewhere in Somersetshire, as a cook, which had been his business before his misfortune, so that I heard no more of him. There was every reason to expect, from the progress which had been made by six applications, that I should have been able to have given a very good account of the case long before this period; but there is the less cause of regret, as I have it in my power to lay before your readers the following still more apposite case.

October 12, 1802. Richard Jacques, No. 6, Little Mary-le-bone Street, about eighteen months ago, lost the use of both his hands; of the two, the right seemed most affected; on the back of it there was a large ganglion-like tumour, which, with other circumstances, made the case resemble that species of paralysis which succeeds the painter's colic; though we could not discover that he had been exposed to the influence of lead.

He had been a patient of the Mary-le-bone Infirmary for six months, and of the Middlesex Hospital for three; of course, all the common remedies had been skilfully applied, but unfortunately had not produced the desired effect. He was not, at the above date, any more than he had been during the whole existence of the affection, able to exercise his occupation, that of a shoemaker. In the two months preceding the above mentioned date, the application had been made two and twenty times. He received no great benefit with respect to motion for the first twelve times; though the tumour on the back of the right hand had been gradually decreasing. At this period, however, different sensations took place. The susceptibility of the parts had become much more considerable. He was seized with *cholera morbus*, a disease at that time prevalent; after which the bowels, hitherto in a torpid state, became more naturally irritable. His countenance lost somewhat of its leaden paleness. The secretion of bile seemed evidently evidently more considerable. A degree of painful itching in the affected parts, and considerable fever took place, exactly as happened in the case of Rouneberg; which, as in that case, was the cause of suspending the application for ten days. The amendment, however, was such as to make the poor fellow very solicitous to persevere in the use of the means. He has now so far recovered the use of his hands as to be able to make two pair of women's shoes per day, and there is every reason to hope he will be able eventually to finish three pair in the same space of time; which he was in the habit of doing before he had been seized with the complaint. I am, &c.

RALPH BLEGBOROUGH.

*No.* 330, *Oxford Street, Jan.* 18, 1803.

